# A Call to the Senses: The Community Approach

**DOI:** 10.3389/fped.2017.00164

**Published:** 2017-07-28

**Authors:** Michele Y. F. Kong

**Affiliations:** ^1^Pediatric Critical Care Medicine, The University of Alabama at Birmingham, Birmingham, AL, United States

**Keywords:** autism spectrum disorders, sensory sensitivity, pediatrics, sensory regulation, family

Inconsolable screaming when touched. Intolerance to bright lights and sudden noise. Frequent meltdowns for no apparent reason. Excessive hand wringing and verbal outburst of sounds. This was our introduction to autism when our 4-year-old son was diagnosed. We quickly learned that a seemingly trivial input (for instance when the doorbell rang) often would lead to an exaggerated response—him having a full-blown meltdown. We found ourselves avoiding public spaces. Eating out, going to movies, or attending sporting events became a distant memory. It was simply too hard, and soon without realizing it, we were in social isolation.

For children starting out in life, well before verbal skills develop, the processing of sensory information represents one of the key sources for adapting effectively to the world. It allows them to register input from the environment and then generates ideas and behaviors based on that input. When sensory processing is atypical, it can be highly disruptive, manifesting itself as emotional or behavioral problems. Unlike blindness or deafness, where sensory information is not received, in these cases information is received but the brain processes that information in ways that result in distress and confusion. This is especially true when more than one sensory modality is involved and when the event is unexpected. For these children, the abnormal perceptions mean that their inner experience is one, not of clarity, but of chaos.

In recent years, we are beginning to have an increased understanding of atypical sensory responses seen in children with autism spectrum disorder (ASD) (1). They often have sensory abnormalities that can range from oversensitivity to vision, audition, and tactile input (e.g., discomfort to bright lights, loud noise, and tags in shirts) to hyposensitivity in other realms (e.g., failing to perceive painful stimuli). They may have a disturbed sensitivity to proprioceptive and vestibular inputs, which can lead to intense hugging and crashing into walls, or the affinity for swinging and rocking (2). Even when the sensory processing is not disordered, most will have some degree of sensory sensitivity. In 2013, abnormal sensory reactivity was included in the current DSM-5 diagnostic criteria (3), highlighting the increased awareness of this realm in ASD.

Those with well-developed nervous systems can handle incoming sensory information in ways that allow us to focus on what is important, filter out what is unimportant and evaluate the varied input so as to gain an understanding of the situation. Consider for instance, a basketball game where we have the ability to focus on the players and the sequence of the play, despite the loud sounds in the arena, and the flurry of other activities that may be going on simultaneously. For our son, because of his sensory sensitivity, the same situation often was overwhelming. As he walked through the arena, the smell of popcorn, the shouting fan from an aisle away, the yelling from the vendor selling drinks, the bright lights, and the need to wait behind a long line to get to the assigned chair frequently triggered a sensory overload and subsequent meltdown. A recent review by Cascio et al. demonstrated the contribution of sensory processing differences in creating the social deficits as well as highlighting the broad impact of sensory dysregulation in the lives of children with autism (4).

As physicians, we use evidence-based medicine to diagnose, manage, and treat our patients. In this emerging realm of sensory processing in ASD, we are beginning to understand the neural pathways and mechanisms underlying sensory regulation. Recently, Schauder and Bennetto outlined a comprehensive review of the current literature as it relates to sensory dysfunction in ASD (5). However, much remains unknown regarding the impact it has on social isolation. In filling the vacuum, it is critical that we shift our focus to changing how we as a society can adapt to the unique needs of autistic children and lessen the practical challenges of atypical sensory responses that they experience.

This year, “Sesame Street” introduced to the world its first autistic character, Julia (6). In one segment of the show, Julia was shown to cover her ears because of intolerance to loud noise. She also flapped her hands when she was upset. Characters such as Julia will help highlight the real challenges that autistic children face, especially in the realm of having sensory sensitivities. In addition, it is now common to see sensory friendly events being organized for the children and their families. For instance, a theater may decrease the volume or dim the lights for a specific performance. There may even be a specially trained staff at hand in order to deal with any issues that these children may have. The problem is that events, even if there are several events a year, form a minuscule part of the child’s life. The principles of the events should be ones that permeate as much of the child’s experience as possible. What we need is thoughtful analysis of ways that we can restructure aspects of our society so that the children and their families can be included in many more public events at all times.

True inclusion is when the child can be at any public space, in any given time, without being limited to only “special sensory friendly events.” The Quicken Loans Arena has taken the first bold step toward becoming the country’s first sensory inclusive arena (7, 8). One might wonder how a basketball arena can be sensory inclusive—it is without a doubt loud, bright, and potentially overwhelming for the sensory sensitive child. It is not as if we could turn the volume of a game down—after all that is part of the excitement of watching a live game. But it is possible to achieve this goal. The first step involves training of all the personnel and staff on how to recognize children with sensory sensitivities, how to use the best methods to engage and communicate with them, as well as how to implement de-escalation and calming techniques when a child is having a meltdown. In addition, the children are provided with sensory bags that contain noise canceling headphones, weighted lap pads, fidget tools, and sensory cue cards. A different entrance is used in order to facilitate the entry process and to allow for exit/re-entry when the child is overwhelmed. With these simple yet impactful modifications, the arena is setting the gold standard of what it means to be sensory inclusive. Similarly, experience at the Birmingham Zoo provides evidence that public spaces can be structured to achieve this goal (9).

What has been done to date is only one small piece of the puzzle. But it can serve as a template for enabling us to alter our mindset to determine how other contexts can be modified so that integration of the children and their families into the community becomes a reality. While we continue to work out the precise links between the brain and sensory processing in autism, it is imperative that we modify the practices of our communities in ways that will enable the children to become full members of our society. We have certainly made progress with increasing autism awareness and acceptance, but more needs to be done. Just as Christine Ferraro said “I would love her to be not Julia, the kid on ‘Sesame Street’ who has autism. I would like her to be just Julia” (10). All our children should be able to enjoy what society has to offer with full accessibility without being limited to “special sensory or autism friendly events.”

## Author Contributions

MK contributed to the conception and writing of this manuscript.

## Conflict of Interest Statement

The author declares that the research was conducted in the absence of any commercial or financial relationships that could be construed as a potential conflict of interest.
